# Society Meetings

**Published:** 1910-05

**Authors:** 


### SOCIETY MEETINGS.

The Homeopathic Medical Society of Western New York held its 26th annual meeting at The Genesee Hotel, April 15, 1910. Papers at the afternoon session were read by R. Montford Schley and F. Park Lewis of Buffalo, A. Jerome Roberts of Jamestown, A. Wilson Dods of Fredonia, E. B. Nash of Port Dickinson, John M. Lee and C. T. Graham of Rochester and Joseph Rieger of Dunkirk.

The officers elected were Charles N. Sumner, Rochester, president; George T. Moseley, Buffalo, vice-president; H. W. Hoyt, Rochester, second vice-president; and R. M. Schley, Buffalo, secretary and treasurer.

The board of censors is composed of these physicians: G. S. Price of Fairport, John Baker of Batavia, William G. Prish of Fredonia, H. G. Shepard of Rochester, W. Lewis Wilson of Niagara Falls.

---

The Medical Society of the County of Erie held its regular meeting Monday, April 18, 1910, at 8.15 P. M., at the Buffalo Library Building. Program: regular business, "The Importance of Urinary Examination in Children," William Irving Thornton; "Inflammation of Verumontanum with Exhibition of Instruments and Cases," James A. Gardner. Intermission. "Exhibition of Fillaria Sanguinis Hominis," J. H. Potter and L. S. Beals; "Routine Fecal Examination," A. L. Benedict. Grover W. Wende is the president and Franklin C. Gram is the secretary.

---

The Buffalo Academy of Medicine held meetings during the month of April, 1910, as follows:

*Special Meeting.*—Tuesday evening, April 5. This meeting was called to receive nominations for president. Program: (a) The Vaccines in the Operative Treatment of Tuberculous Joints, William Ward Plummer; (b) Anti-Typhoid Vaccines, William G. Bissell. The discussion was opened by Norman K. McLeod.

*Section of Medicine.*—Tuesday evening, April 12. Program: (a) Social Service in Hospital Work, Richard C. Cabot, Boston, Mass., Clinical Professor of Medicine in Harvard Medical School; (b) Same Title, Charles P. Emerson, Clifton Springs, N. Y., Superintendent of Clifton Springs Sanitarium. Discussion was opened by Charles G. Stockton.

*Section of Obstetrics.*—Tuesday evening, April 19. Program: (a) A Plea for Delivering Our Maternity Patients in Hospitals, L. Schroetter; (b) Some Thoughts about Retroversion, C. C. Frederick. Discussion was opened by P. W. VanPeyma.

*Section on Pathology.*—Tuesday evening, April 26. Program: Toxemias of Pregnancy, by J. Whitridge Williams, Baltimore, Md.

---

The Rochester Academy of Medicine held meetings during the month of March, 1910, at the Hotel Seneca, as follows:

*General Medicine.*—Wednesday evening, April 6. Program: Vaccine Therapy, C. O. Boswell.

*Surgery.*—Wednesday evening, April 13. Program: The General Surgeon's Plastic Surgery, John F. W. Whitbeck.

*Obstetrics.*—Wednesday evening, April 20. Program: Infections of the Female Genital Tract, Wm. D. Johnson, Batavia. Discussion opened by Thomas Jameson.

---

The American Medical Association will meet at Saint Louis, June 7-10, 1910, under the presidency of Dr. William H. Welch, of Baltimore. Dr. Walter B. Dorsett, Linmar Building, Saint Louis, is the chairman of the Committee of Arrangements.

---

The American Proctologic Society will hold its twelfth annual meeting at St. Louis, Mo., June 6 and 7, 1910. Headquarters and place of meeting, Planters' Hotel, Fourth and Pine. The profession is cordially invited to attend all meetings.

